# Dynamic recognition and linkage specificity in K63 di-ubiquitin and TAB2 NZF domain complex

**DOI:** 10.1038/s41598-018-34605-2

**Published:** 2018-11-07

**Authors:** Kei Moritsugu, Hafumi Nishi, Keiichi Inariyama, Masanori Kobayashi, Akinori Kidera

**Affiliations:** 1 Graduate School of Medical Life Science, Yokohama City University, 1-7-29 Suehiro-cho, Tsurumi-ku, Yokohama, 230-0045 Japan; 2 Present Address: Graduate School of Information Sciences, Tohoku University, 6-3-09 Aramaki-aza Aoba, Aoba-ku, Sendai, Miyagi 980-8579 Japan

## Abstract

Poly-ubiquitin (poly-Ub) is involved in various cellular processes through the linkage-specific recognition of Ub-binding domains (UBD). In this study, using molecular dynamics (MD) simulation together with an enhanced sampling method, we demonstrated that K63-linked di-Ub recognizes the NZF domain of TAB2, a zinc finger UBD, in an ensemble of highly dynamic structures that form from the weak interactions between UBD and the flexible linker connecting the two Ubs. However, the K63 di-Ub/TAB2 NZF complex showed a much more compact and stable ensemble than the non-native complexes, linear di-Ub/TAB2 NZF and K33 di-Ub/TAB2 NZF, that were modeled from linear di-Ub/HOIL-1L NZF and K33 di-Ub/TRABID NZF1, respectively. We further demonstrated the importance of the length and position of the Ub-Ub linker in the results of MD simulations of K63 di-Ub/TAB2 NZF by changing the Ub linkage from the native K63 to four different non-native linkages, linear, K6, K11, and K48, while maintaining inter-molecular contacts in the native complex. No systems with non-native linkage maintained the native binding configuration. These simulation results provide an atomistic picture of the linkage specific recognition of poly-Ubs leading to the biological functions such as cellular colocalization of various component proteins in the signal transduction pathways.

## Introduction

Versatile molecular recognition of poly-ubiquitin (poly-Ub) chains results from their structural variation in the long flexible Ub-Ub linker (Ub 71–76 + side-chain of Lys) with eight different linkage types connecting distal and proximal Ubs through one of the seven Lys residues or amino terminus. These distinct linkages of poly-Ub chains underlie the binding specificity of various ubiquitin-binding domains (UBDs)^1,2^. A variety of UBDs bound to poly-Ub leads to a multitude of cellular functions in which poly-Ub scaffolds colocalize with specific proteins by binding their component UBDs and integrating them into a wide array of cellular functions, depending on the poly-Ub linkage types^3–5^.

The most representative case is activation of the nuclear factor (NF)-κB transcription factor. K63-linked and linear poly-Ub chains function as a scaffold to colocalize upstream and downstream kinases, transforming growth factor-β-activated kinase 1 (TAK1), and IκB kinase (IKK)^6–8^. TAK1 binds to K63 poly-Ub via the Npl4 zinc finger (NZF) domain of the adaptor TAK1-binding protein 2 (TAB2) or TAB3^9–11^. In contrast, IKK attaches linear poly-Ub through the UBAN domain of the regulator protein NF-κB essential modulator^12^. Because of the colocalization of these kinases on poly-Ub chains, IKKβ, a component of IKK, is phosphorylated through TAK1 and activates downstream signaling.

The structural basis of the specific recognition of UBDs by poly-Ub was determined in crystallographic studies of UBD bound to di-Ub as a minimal polyubiquitylation unit, which constitutes the key element of poly-Ub recognition. The recognition modes observed in the crystal structures provide important information regarding the mechanism of how linkage specificity is achieved^1,2,12^. The crystal structures reveal a common binding mode in which non-interacting proximal and distal Ubs, connected by the long Ub-Ub linker, form a bidentate interaction with a UBD^12^.

In addition to crystallographic studies, extensive experimental studies have been conducted. Binding assay experiments clearly revealed the effects of multivalent binding, or “avidity”, at proximal and distal Ubs; the dissociation constants, *K*_d_, of di-Ub/UBD complexes were mostly of the order of 1–10 μM, which are smaller by an order of magnitude than those for mono-Ub complexes (*K*_d_ values are typically >100 μM)^13^. The multivalent effects have mainly been attributed to the increase in the local density of Ub around the UBD because of colocalization of the two binding sites^14–16^. The multivalent effects in di-Ub suggest the following process for di-Ub/UBD recognition: the UBD repeatedly dissociates and rebinds through the colocalization because of the weak binding to each moiety. These association/dissociation motions occur on flexible Ub moieties. Therefore, the binding mode is not unique but rather broadly distributed, and these features are collectively referred to as “dynamic recognition.”

**Figure 1:**
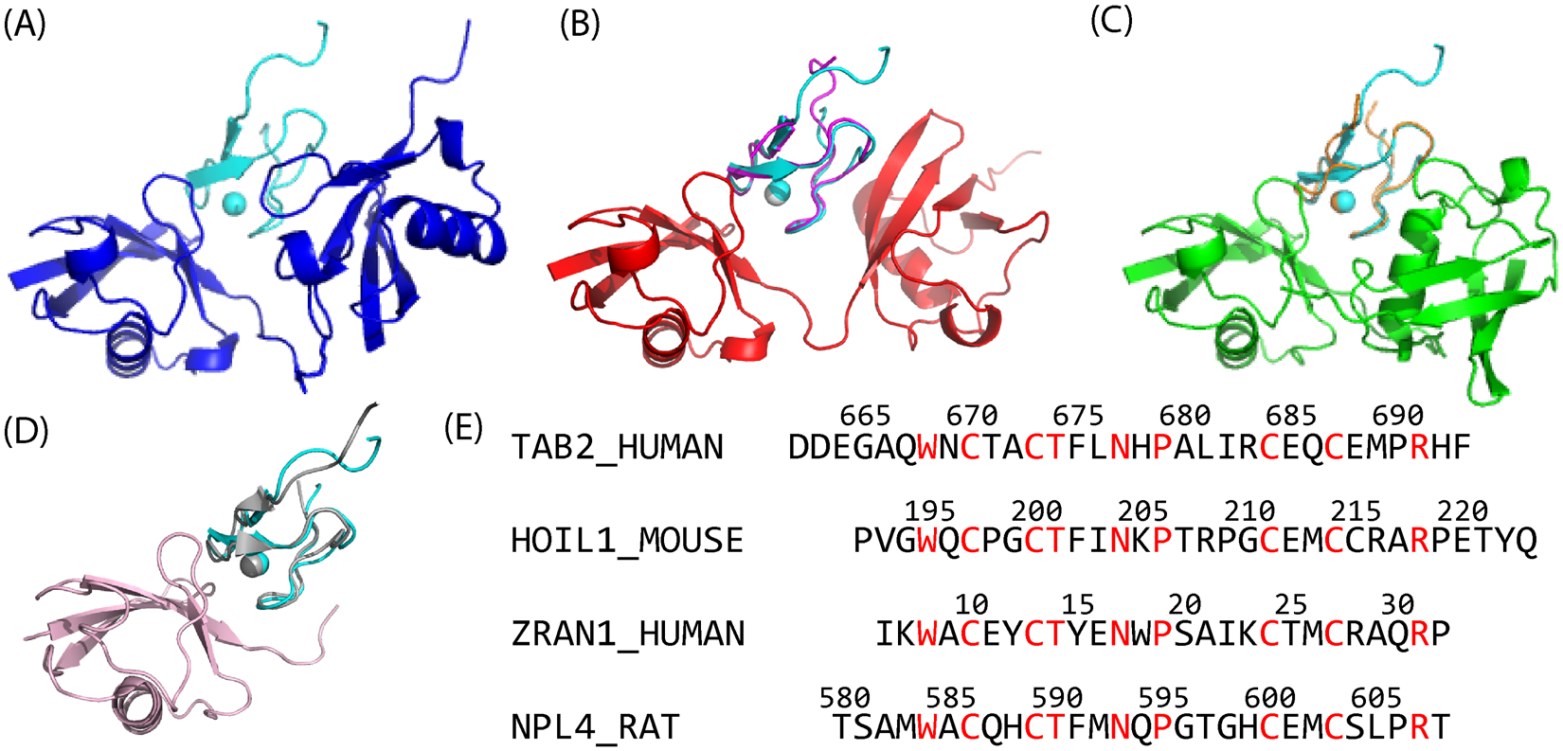
Crystal structures of the di-Ub/NZF complexes and sequence alignment of the NZF domains. (**A**) K63 di-Ub/TAB2 NZF (PDB: 2wwz). (**B**) Linear di-Ub/HOIL-1L NZF Δtail (PDB: 3b0a). Here, Δtail indicates that the NZF domain does not contain the C-terminal helix of HOIL-1L (224–249). (**C**) K33 di-Ub/TRABID NZF1 (PDB: 5af6). (**D**) Mono Ub/NPL4 NZF (PDB: 1q5w). In (**B**–**D**), TAB2 NZF (cyan) is drawn after superimposition to the core region (1–70) of the distal Ub (left moiety of di-Ub) or mono-Ub. (**E**) The sequences of the NZF domains used in this study were aligned. ZRAN1 corresponds to TRABID. Red characters indicate the amino acids which are conserved in all four proteins.

In this study, we evaluated the structural basis of dynamic recognition of UBD by di-Ub and how linkage specificity is achieved in this dynamic recognition through molecular dynamics (MD) simulations. We chose the K63 di-Ub/TAB2 NZF complex mentioned above as the target system (Fig. 1). The NZF domain is a family of small zinc finger domains frequently observed in complex with poly-Ub, whose structural and biochemical information is abundant; the structures of di-Ub complexes were solved for TAB2 (K63), TAB3 (K63), HOIL-1L (linear), and TRABID (K29 and K33), with the names in parentheses indicating the linkage type specifically bound by di-Ub (Fig. 1)^9,10,17–19^.

First, to characterize the dynamic recognition in K63 di-Ub/TAB2 NZF, we evaluated the free energy landscape of the intermolecular interactions, from tightly bound states to deformed states, using an enhanced sampling method named as multiscale enhanced sampling (MSES) (see Methods for details)^20–22^. The landscape of the interactions revealed an ensemble of various binding modes. Second, we determined the linkage specificity of TAB2 NZF from two perspectives: the interacting surface and connectivity of the Ub-Ub linker. The stability of the interactions with different interfaces was examined by simulations of linear di-Ub/TAB2 NZF and K33 di-Ub/TAB2 NZF, of which initial structures for the simulations were obtained by homology modeling based on the linear di-Ub/HOIL-1L NZF and K33 di-Ub/TRABID NZF1 crystal structures^17,19^. Next, we considered the complex structures while maintaining the interacting surface of K63 di-Ub/TAB2 NZF but changing the connectivity of the Ub-Ub linker to those of linear, K6, K11, and K48. These simulations of the complexes with artificial linkage revealed the structural importance of the Ub-Ub linker in linkage specificity.

## Results and Discussion

### Free energy landscape of the interactions between K63 di-Ub and TAB2 NZF

In the preliminary runs of five 150-ns MD simulations for the K63 di-Ub/TAB2 NZF complex in solution starting from the crystal structure (PDB: 2wwz)^9^, the complex exhibited marginal stability (average Cα root-mean-square displacement (Cα RMSD) for the core region of the complex (di-Ub: 1–70 and TAB2 NZF: 669–691) was 2.2 ± 1.3 Å during 20–150 ns, Fig. S1A). The MD simulations for another crystal structures of K63 di-Ub/TAB2 NZF (PDB: 3a9j)^10^ also showed marginal stability, with an average Cα RMSD of 4.2 ± 2.9 Å (Fig. S1B; comparison of the simulation results are discussed below). The marginal stability of the complex suggests dynamic recognition between the di-Ub and the NZF domain mentioned above, and thus we surveyed a sufficiently large configurational space of the complex to evaluate the free energy landscape. We employed the MSES simulation^20–22^, which yields the canonical distribution of the large configurational space of proteins using a multiscale simulation based on an all-atom model in water coupled with a coarse-grained model^23–26^.

**Figure 2:**
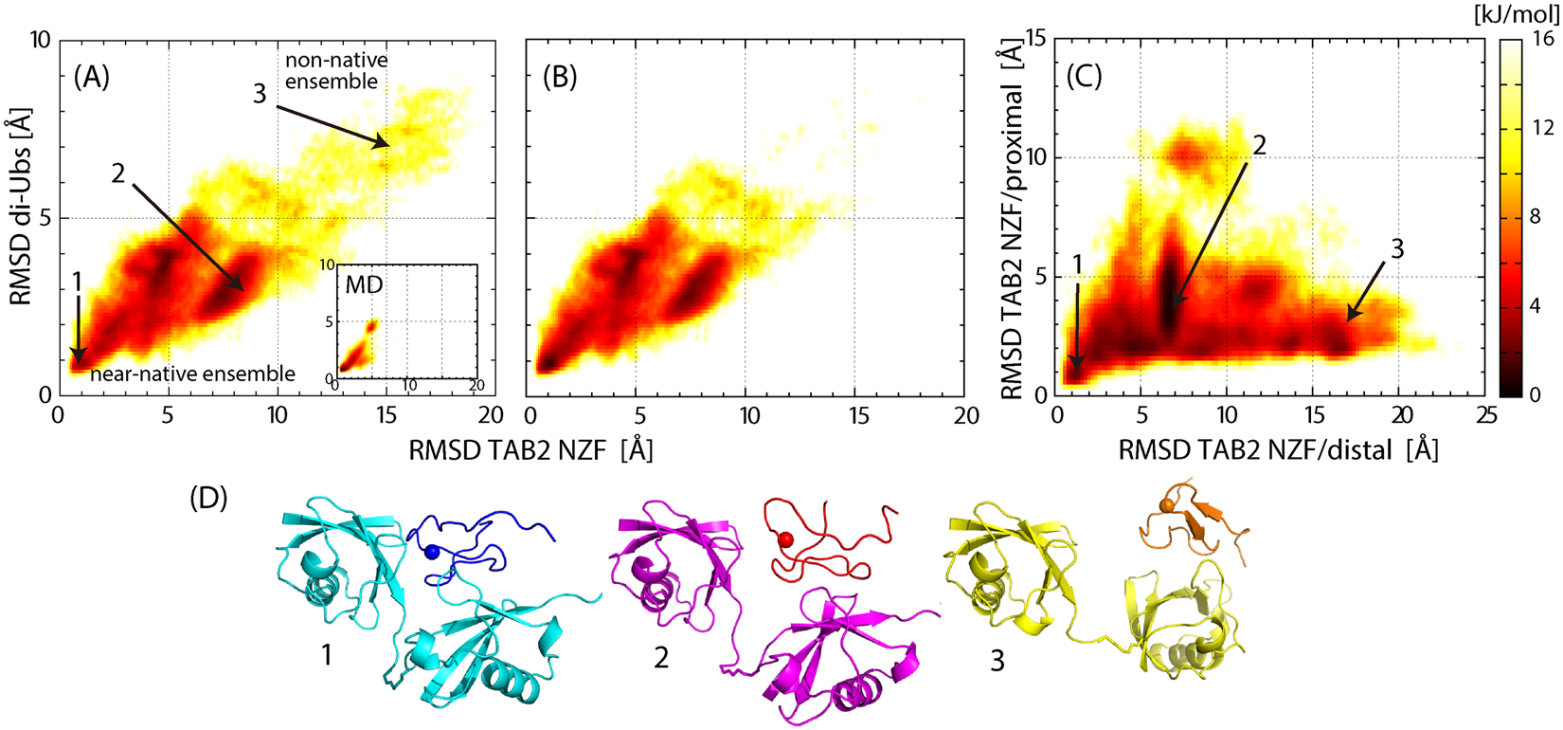
FES of K63 di-Ub/TAB2 NZF. (**A**) FES plotted on a two-dimensional space, Cα RMSD values of TAB2 NZF, and di-Ub from the crystal structure (PDB: 2wwz) after superimposing Cα atoms of the core regions of di-Ub (1–70). The inset is the same plot but calculated from five 150-ns MD simulations (Fig. S1A). The lower free energy region (<10 KJ/mol) is named as the “near-native” ensemble, while the higher free energy region (>10 KJ/mol) is named as the “non-native” ensemble. (**B**) The FES for the subset with the condition that TAB2 NZF contacts (any non-hydrogen atom pairs whose distance is less than 4 Å) both distal and proximal Ubs. (**C**) The same as (**A**) but plotted on the two Cα RMSD values of TAB2 NZF, one superimposing the distal Ub (1–70) and the other superimposing the proximal Ub (1–70). (**D**) Three representative structures of the complex whose position in the FES are indicated by the numbers in (**A**,**C**).

The MSES simulation was successfully conducted for the K63 di-Ub/TAB2 NZF complex, which showed a large acceptance ratio (average of 0.32) in the replica exchange operation and convergence of the derived structural ensemble (Fig. S2). In Fig. 2, the results of MSES simulation are presented in the form of free energy surfaces (FES) together with representative structures. The FES, described by the Cα RMSD values of the core region of the complex defined above, primarily represents the rigid body (translational and rotational) motions of the three component molecules, the two Ubs and the NZF domain, since the fluctuations of the core are dominated by inter-molecular motions; the intra-molecular RMSD values within the core were 0.75 Å and 1.5 Å for Ub and TAB2 NZF, respectively, which amount about 20% of the total RMSD. Hereinafter, the RMSD values in the text and figures indicate those for the core regions of the complex. Comparison with the conventional MD simulation (inset of Fig. 2A; results of five 150-ns MD runs in Fig. S1A are aggregated on the same two-dimensional space) indicated that the MSES simulation enhanced the sampled area enough to evaluate the FES of the complex from the tightly bound structure to nearly dissociated structures. The FES was clearly divided into two parts (Fig. 2B): a highly populated region including the crystal structure (free energy <10 KJ/mol; referred to as the “near-native” ensemble) and another less populated large RMSD region (“non-native” ensemble). When a subset was selected from the whole ensemble under the condition that the NZF domain contacted both distal and proximal Ubs, the subset ensemble was found to overlap well with the near-native ensemble (Fig. 2B). This overlap indicates that the near-native ensemble is characterized as *bidentate binding* of the NZF domain by two Ub moieties, and its large RMSD ranges, up to 10 Å for NZF and up to 5 Å for di-Ub, indicate dynamic recognition consisting of various binding modes. In contrast, the non-native ensemble corresponded to the complex structures whose NZF domain was bound only at a single Ub moiety, and it was thus characterized as *monodentate binding*. The FES clearly showed that the bidentate interactions in the near-native ensemble were distinctly more stable (more populated) than in monodentate binding. However, it is noted that the FES exhibits a rather smooth surface without any significant barrier between the two ensembles.

Dynamic recognition of the K63 di-Ub/TAB2 NZF complex illustrated by FES was considered to be mainly related to low intrinsic affinity between a Ub moiety and TAB2 NZF (experimental *K*_d_ values = several hundred μM or higher)^9,10,27^, as well as intrinsic flexibility of di-Ub connected by the long Ub-Ub linker. The flexibility of K63 di-Ub has been demonstrated in various solution experiments of free K63 poly-Ub in solution, including nuclear magnetic resonance, Fluorescence resonance energy transfer, and small-angle X-ray scattering^28–30^. Despite these causes of instability, the near-native ensemble appeared to form a distinct cluster of low-free energy structures (Fig. 2A).

### Structural origin of dynamic recognition

**Figure 3:**
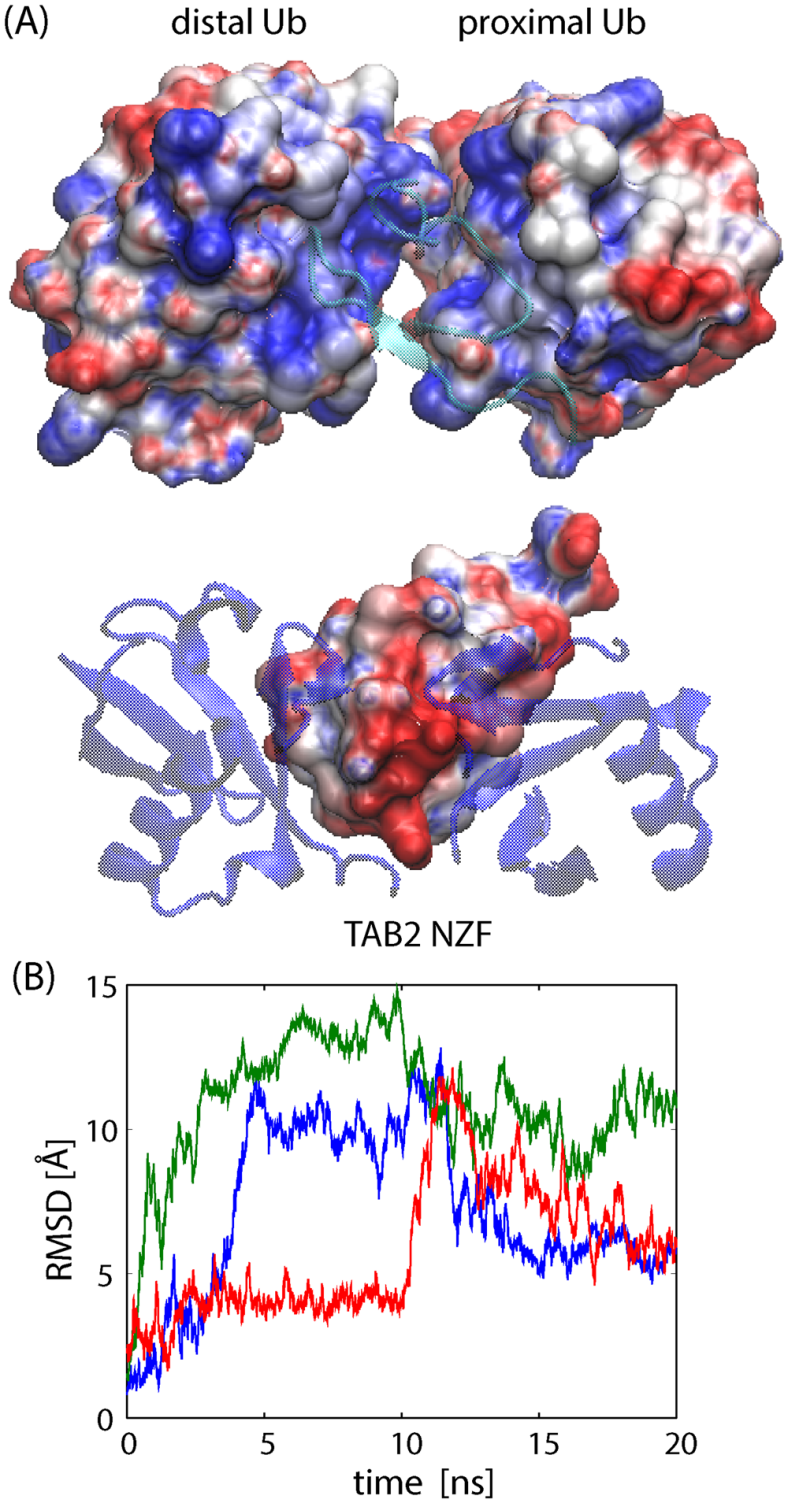
Electrostatic interactions between K63 di-UB and TAB2 NZF. (**A**) Surfaces of the electrostatic potential of K63 di-Ub without TAB2 NZF and isolated TAB2 NZF, drawn by VMD^47^. (**B**) Time courses of Cα RMSD for three MD simulations (colored in red, blue, and green) of free K63 di-Ub (core regions, 1–70) starting from the crystal structure of the complex after removing TAB2 NZF.

The cause of dynamic recognition allowing various binding modes in the near-native ensemble can primarily be attributed to the electrostatic interactions between the Ub moieties and TAB2 NZF. Figure 3A shows that the basic interacting surface of K63-linked Ub sandwiches the acidic surface of TAB2 NZF. These long-range electrostatic interactions are used to stabilize the complex in a variety of bidentate binding modes within the near-native ensemble. The importance of the electrostatic interactions was also observed in the MD simulations of free K63 di-Ub in the compact form, which was modeled by removing TAB2 NZF from the crystal structure of the complex (Fig. 3B). The compact form was not maintained and largely changed its structure within 5–10 ns because of electrostatic repulsion between basic residues. Comparison with the results of MD simulations for free and compact forms of linear di-Ub and K33 di-Ub confirmed that electrostatic interactions significantly contributed to stability (Figs S3 and S4). Linear di-Ub immediately extends the structure because of electrostatic repulsion as in K63 di-Ub. In contrast, K33 di-Ub also extends the structure but not as quickly as in K63 and linear di-Ubs, mainly because of the absence of strong electrostatic repulsion. These results are consistent with those of solution experiments^19,28–30^.

We further examined the detailed inter-molecular interactions between K63 di-Ub and TAB2 NZF. Table S1 summarizes the inter-molecular contacts with a high probability of occurrence observed in MSES simulation, including numerous non-crystal contacts (not observed in the crystal structure), thus indicating dynamic recognition consisting of various binding modes. Among the variety of binding modes, the crystal structure showed the largest number of intermolecular contacts and thus the minimum energy structure dominated at a low temperature at which the crystal structure was determined (Fig. S5). Thus, dynamic recognition is not simply a loosely bound complex, but characterized by a funnel-shaped ensemble centered on the crystal structure. As shown in Table S1, proximal Ub binds more tightly than distal Ub. When the relative position of TAB2 NZF to distal Ub and proximal Ub were calculated in the FES (see Fig. 2C), it was confirmed that the NZF domain maintained the interactions to the proximal Ub throughout the whole ensemble, but not to the distal Ub. This indicates that the non-native ensemble in Fig. 2A exhibits monodentate binding to proximal Ub (Fig. S5).

Recognition of the UBDs by Ub is commonly described with the hydrophobic patches F4, I36, and I44^2^. In the crystal structure of K63 di-Ub/TAB2 NZF, the two I44-patches (L8, I44, H68, and V70) of the distal and proximal Ubs sandwich the NZF domain^9,10^. The binding mode at the distal Ub is shared by complexes of the di-Ub with different linkages, linear di-Ub/HOIL-1L NZF and K33 (K29) di-Ub/TRABID NZF1, as well as in mono-Ub/Npl4 NZF (PDB: 1q5w)^27^, whose positions of the distal Ub and NZF domain resemble each other (Fig. 1A–D). The common binding form in distal Ub means that linkage-specific recognition occurs in the proximal Ub, and that stability at the proximal Ub determines linkage specificity. In fact, a mutation in TAB2 NZF, Q686M, stabilizes the interaction with distal Ub, making the recognition linkage-independent^10,18^. In the next paragraph, we examined a factor making affinity to the proximal Ub higher than that of the distal Ub.

**Figure 4:**
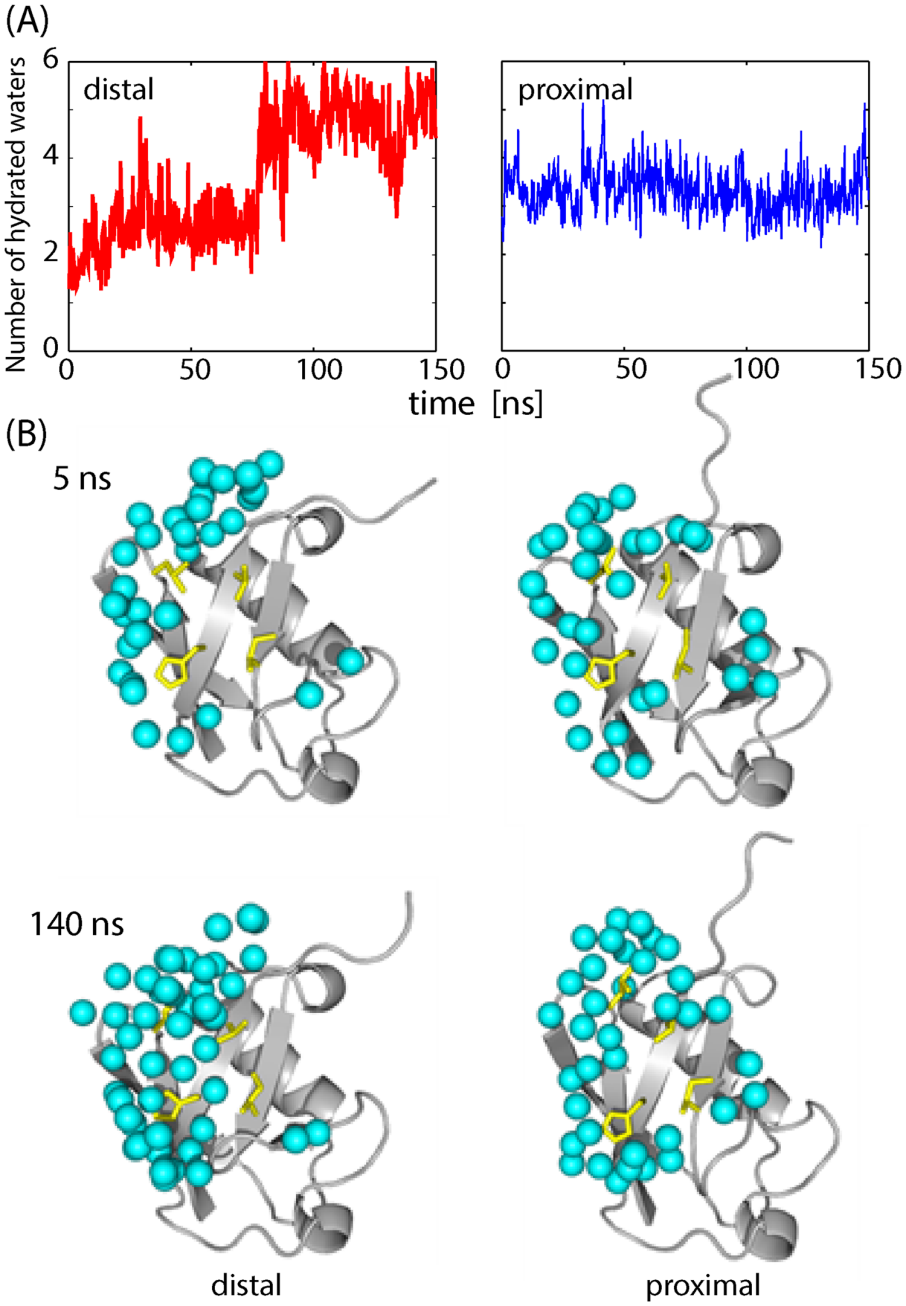
Hydration of the interface between di-Ub and TAB2 NZF. (**A**) Number of hydrated water molecules found on the interface (waters within 4 Å of the protein interface). The left and right figures are for the distal Ub and proximal Ub, respectively. (**B**) The snapshots of interfacial waters for the distal Ub (left) and proximal Ub (right) after 5 ns (upper) and 140 ns (lower) during MD simulation. The water molecules are depicted by cyan spheres and the four residues of the I44 patch (L8, I44, H68, and V70) are drawn as yellow sticks.

Table S1 shows that the contacts containing the I44-patch were nearly broken at the distal Ub, but retained at the proximal Ub. The dissociation of the hydrophobic I44-patch at the distal Ub was caused by the invasion of water molecules into the hydrophobic region as depicted in Fig. 4 (the observation in one of the MD simulations whose RMSD value reached 5 Å after 150 ns, Fig. S1A). In the initial stage of the simulation close to the crystal structure, the patch of the distal Ub had a lower level of hydration, or stronger hydrophobic interactions, compared to that of the proximal Ub (Fig. 4). However, after 80 ns, the hydrophobic surface of the distal Ub was broken, while that of the proximal Ub maintained the hydration level. Binding at the hydrophobic patch is rather short-range, as the distance of the interfacial solvation/desolvation determines the interaction range, and thus hydrophobic interactions at a mobile interface may be susceptible to large thermal fluctuations of the component molecules. These observations suggest that the proximal Ub has another source of a longer-range interaction to stabilize the interface other than the I44-patches.

Table S1 also indicates that the polar contacts of the distal Ub are hydrogen bonds, whereas those of the proximal Ub are mostly salt bridges, where the acidic cluster of the N-terminus of TAB2 NZF (662–664, DDE, Fig. S6) plays an important role in maintaining the interaction with proximal Ub. As shown above, the MD simulations starting from another crystal structure of the K63 di-Ub/TAB2 NZF complex (PDB: 3a9j) revealed larger instability (Cα RMSD from the crystal structure 4.2 ± 2.9 Å) than simulations starting from 2wwz (Cα RMSD from the crystal structure 2.2 ± 1.3 Å) (Figs S1A and B). The difference between the two simulation systems lies only at the N-terminal acidic cluster of TAB2 NZF (662–664); the NZF domain in 3a9j lacks the acidic cluster (665–693), while that of 2wwz contains the acidic cluster (662–693). Other than this difference, these two systems contained identical amino acids (although they differ in different organisms, human (2wwz) and mouse (3a9j)), with very similar initial structures (Cα RMSD between 3a9j and 2wwz is 0.74 Å). The experimental data also suggest the contribution of the acidic cluster: *K*_d_ = 8 μM for K63 di-Ub/TAB2 NZF (663–693)^9^, while *K*_d_ = 64.2 μM for K63 di-Ub/TAB2 NZF (665–693)^10^. These results confirm the significant contribution of the N-terminal acidic cluster to K63 di-Ub/TAB2 NZF stability.

**Figure 5:**
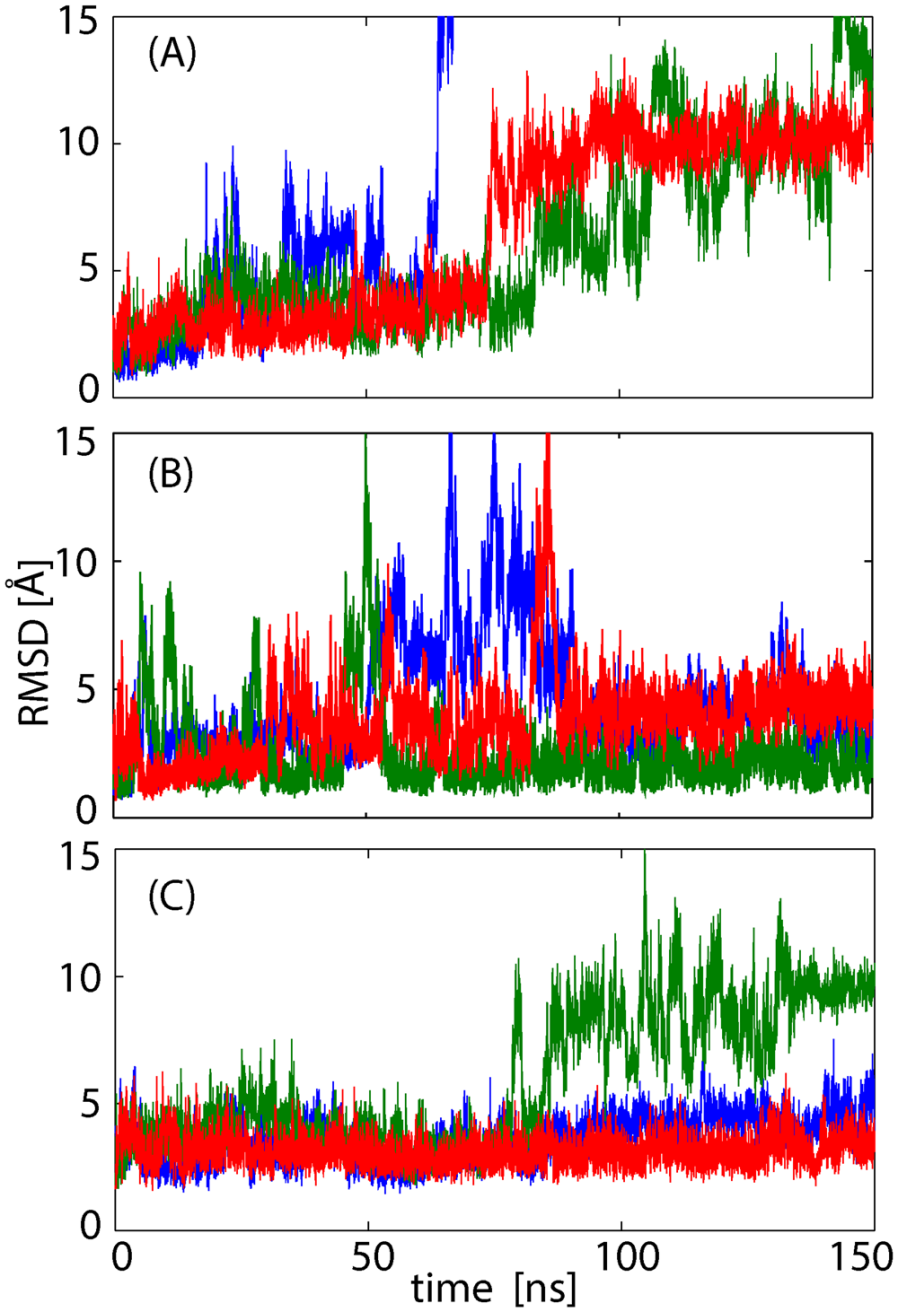
MD simulations of mono-Ub/TAB2 NZF. (**A**) Time courses of Cα RMSD values for three 150-ns MD simulations (colored in red, blue, and green) from the crystal structure of distal Ub/TAB2 NZF (PDB: 2wwz as the reference structure). The blue curve overshoots the upper range of the figure. (**B**) Those of proximal Ub/TAB2 NZF. (**C**) Those of mono-Ub/Npl4 NZF (PDB: 1q5w).

To compare the affinities of distal Ub and proximal Ub to TAB2 NZF, we performed three additional MD simulations for the mono-Ub/TAB2 NZF (662–693) complexes: (1) The complex which binds only at the surface of the distal Ub was modeled by removing the proximal Ub from the complex (named as the distal Ub type complex). (2) The proximal Ub type complex was modeled by removing the distal Ub from the complex. (3) Mono-Ub/Npl4 NZF (PDB: 1q5w)^27^ was also studied as a stable reference system, which is at present the only crystal structure solved for the mono-Ub/NZF complex (*K*_d_ value is 126 μM)^27^. The results are shown in Fig. 5. As expected, the proximal Ub type complex, which interacts with the N-terminal acidic cluster, was much more stable than the distal Ub type complex and showed a similar level of stability as the mono-Ub/Npl4 NZF. In the proximal Ub type complex, polar contacts were found mostly in the acidic cluster as in the di-Ub complex (Table S4). As shown in the sequence alignment of the NZF domains of TAB2 and TAB3 (Fig. S6), the acidic cluster of the NZF domain was conserved in the sequences from human TAB2 to *Xenopus* TAB3 (sequence identity of 36%). The experimental *K*_d_ values revealed that the affinity between mono-Ub and TAB2 NZF increased with the number of acidic residues at the N-terminus of the NZF domain; *K*_d_ = 275 μM for NZF (663–693, two acidic residues)^9^, *K*_d_ = 338 μM for NZF (664–693, one acidic residue)^27^, and *K*_d_ = 918 μM for NZF (665–693, no acidic residues)^17^. According to these experimental data and simulation results obtained above, the electrostatic interactions with the acidic cluster determine the recognition between di-Ub and TAB2/TAB3 NZF to allow linkage-specific recognition of NZFs by the proximal Ub of K63 di-Ub. Additionally, mono-Ub/TAB2 NZF^9,17^ may have a major population formed by binding to the proximal Ub, rather than the common binding mode to the distal Ub, when the acidic cluster is present at the N-terminus of the NZF domain.

### Linkage specificity

The linkage specificity of di-Ub has also been described for the hydrophobic patches^2^; specific recognition in di-Ub occurs when the linkage allows hydrophobic patches on the surface of each Ub moiety to correctly face the UBD. Here, we investigated the linkage specificity of TAB2 NZF from two perspectives: the interacting surface and connectivity of the Ub-Ub linker. The interacting surface was studied by MD simulations of the homology models of other di-UB/NZF complexes with different linkages. The connectivity of the Ub-Ub linker was investigated by MD simulations of the complex whose linkages were artificially changed to non-native linkages while TAB2 NZF was kept in the same position as in the native K63 complex.

#### Interacting surface

First, we examined linkage specificity in terms of the interacting surface using simulations of the complexes in which di-Ub with each different linkage recognizes TAB2 NZF using different interfaces, linear di-Ub/TAB2 NZF and K33 di-Ub/TAB2 NZF whose structures were homology-modeled using the crystal structures of linear di-Ub/HOIL-1L NZF Δtail (192–223) and K33 di-Ub/TRABID NZF1, respectively^17,19^. These NZF domains are highly homologous to each other (Fig. 1E), and thus have similar structures or good shape complementarity with di-Ub.

**Figure 6:**
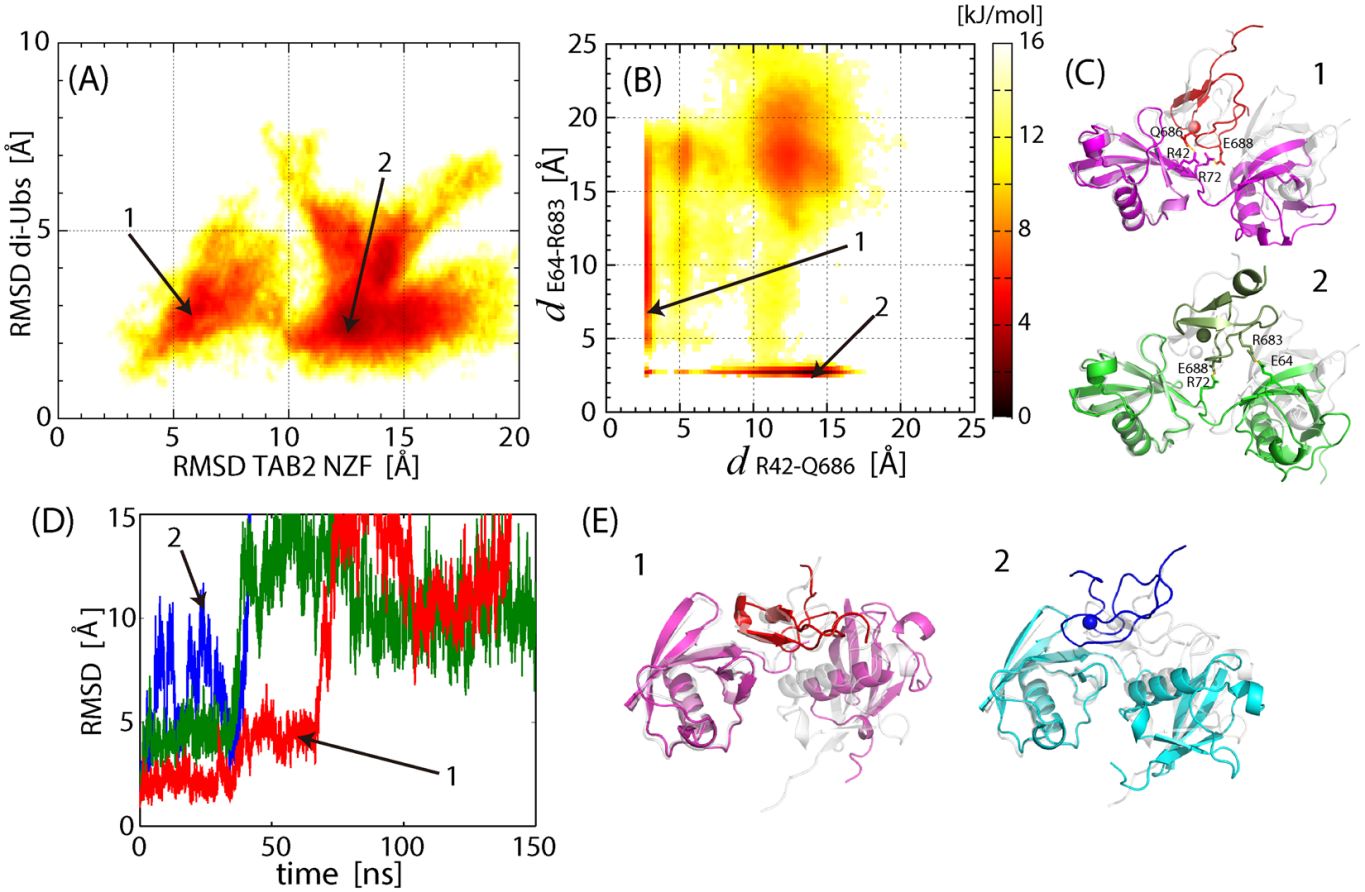
MD simulations of linear di-Ub/TAB2 NZF and K33 di-Ub/TAB2 NZF. (**A**) FES of linear di-Ub/TAB2 NZF obtained in the MSES simulation, plotted on a two-dimensional space, Cα RMSD values of TAB2 NZF and di-Ub from the initial homology model, after superimposing Cα atoms of di-Ub (1–70). (**B**) The same as (**A**), but plotted on the two distances, *d*_R42-Q686_ and *d*_E140-R683_. (**C**) Two representative structures whose positions in the FES are indicated in (**A**,**B**). TAB2 NZF changes the position by rotation while maintaining the salt bridge, R72-E688. (**D**) Time courses of Cα RMSD value of K33 di-Ub/TAB2 NZF from the initial homology model, after superimposing Cα atoms of di-Ub (1–70), obtained in the three 150-ns MD simulations (colored in red, blue, and green). The blue curve overshoots the range of the figure. (**E**) Two representative structures indicated in (**D**).

MSES simulation of the linear di-Ub/TAB2 NZF complex was also successfully performed, as summarized in Fig. S2B (average acceptance ratio for the replica exchange was 0.27). The FES obtained by MSES simulation showed two distinctly separated basins (Fig. 6A). Importantly, neither contained the structure of the initial homology model corresponding to linear di-Ub/HOIL-1L NZF (position of RMSD = 0 Å; see Fig. 6A), indicating that arrangement of the two Ub moieties to bind HOIL-1L NZF is not compatible with the recognition of TAB2 NZF. The NZF domain appears to alternate between the two basins, corresponding to the complexes interacting only with distal Ub (“1” in Fig. 6C; probability of occurrence is 0.24 when the basin is defined by the threshold at RMSD = 9 Å) and only with the proximal Ub (“2”; probability is 0.76), respectively, as shown in the distance profile of Fig. 6B. Separation between the two basins was observed in the inter-molecular contacts, R42 (distal Ub)-Q686 (TAB2 NZF) (probability of occurrence in basin 1 is 0.52) and E64 (proximal UB)-R683 (TAB2 NZF) (probability in basin 2 is 0.53). These two interactions occur exclusively, indicating a discrete transition between these two monodentate binding modes pivoting on the stable salt bridge commonly present in both basins, R72(the Ub-Ub linker)-E688(TAB2 NZF) (probabilities in basins 1 and 2 are 0.68 and 0.63, respectively). The cause of instability in the bidentate interactions in the complex is shown in Table S2. The I44 (distal Ub) and F4 (proximal Ub) patches, stabilizing linear di-Ub/HOIL-1L NZF, lost all stable nonpolar interactions in the complex with TAB2 NZF, whereas polar contacts (or salt bridges) appeared to maintain the complex structure. Notably, however, the four salt bridges in the HOIL-1L NZF complex were all replaced by those with different binding partners (not aligned amino acids) in the TAB2 NZF complex (Fig. S8). These changes in the salt bridges prevented simultaneous binding of TAB2 NZF to linear di-Ub but allowed each monodentate form. This binding mode may represent an unstable limit of dynamic recognition stabilized by the electrostatic interactions.

Additional MD simulations of linear di-Ub/HOIL-1L NZF containing the C-terminal helix (192–249; Fig. S7A) and linear di-Ub/TAB2 NZF (Fig. S7B) were also performed as reference systems based on the native linear complex. We found that removing the C-terminal helix significantly destabilized linear di-Ub/HOIL-1L NZF Δtail as in the experimental *K*_d_ values (17.2 μM for HOIL-1L NZF and 118 μM for HOIL-1L NZF Δtail; Fig. S7B)^17^. However, the artificial complex of linear di-Ub/TAB2 NZF was still more unstable than the native complex with HOIL-1L NZF Δtail (Fig. S7C).

The complex of K33 di-Ub/TAB2 NZF was highly unstable and easily dissociated, as observed in the results of the three 150-ns MD simulations (Fig. 6D,E: therefore, MSES was not successfully performed and instead MD simulations starting with the modeled structure were used). The polar contacts observed in the MD simulations listed in Table S3 were mostly those found at the beginning of the simulation process and not necessarily stable. The native complex, K33 di-Ub/TRABID NZF1, was exceptionally stable because of its dominating nonpolar interactions in such a way as lock-and-key type binding as shown in Table S3 and Fig. S7D, although proximal Ub used none of the hydrophobic patches. Such tight hydrophobic interactions may easily be destabilized by a small number of amino acids changes destroying the shape complementarity. A previous study showed that mutations in TRABID NZF1, Y15F, W18A, S20R, and T25D, corresponding to F675, H678, A680, and E685 in TAB2 NZF, respectively, were sufficient to abolish or significantly weaken binding^19^.

In summary, the linkage specificity in K63 di-Ub/TAB2 NZF is finely encoded in the amino acid sequence of the NZF domain which yields high affinity to the interacting surface given by the K63 di-Ub linkage, and thus changes in the binding surface significantly destabilize the interactions. Each binding surface has its own favorable amino acids coded in the partner UBD. The hydrophobic patches play an important role as the bottom of the funnel-shaped landscape, or as the minimum energy structure ensuring linkage specificity. However, the electrostatic interactions are more important in the dynamic recognition to maintain di-Ub and the UBD close to each other.

#### Connectivity of the Ub-Ub linker

We examine the influence of Ub-Ub linker connectivity on the stability of the di-UB/NZF complex. We performed MD simulations of K63 di-Ub/TAB2 NZF with maintaining the interacting surface but changing the linkage artificially to non-native linkages: linear, K6, K11, and K48. For the other linkages, K27, K29, and K33, the C-terminus of the distal Ub cannot be connected to these lysine side-chains because of strong steric hindrance.

**Figure 7:**
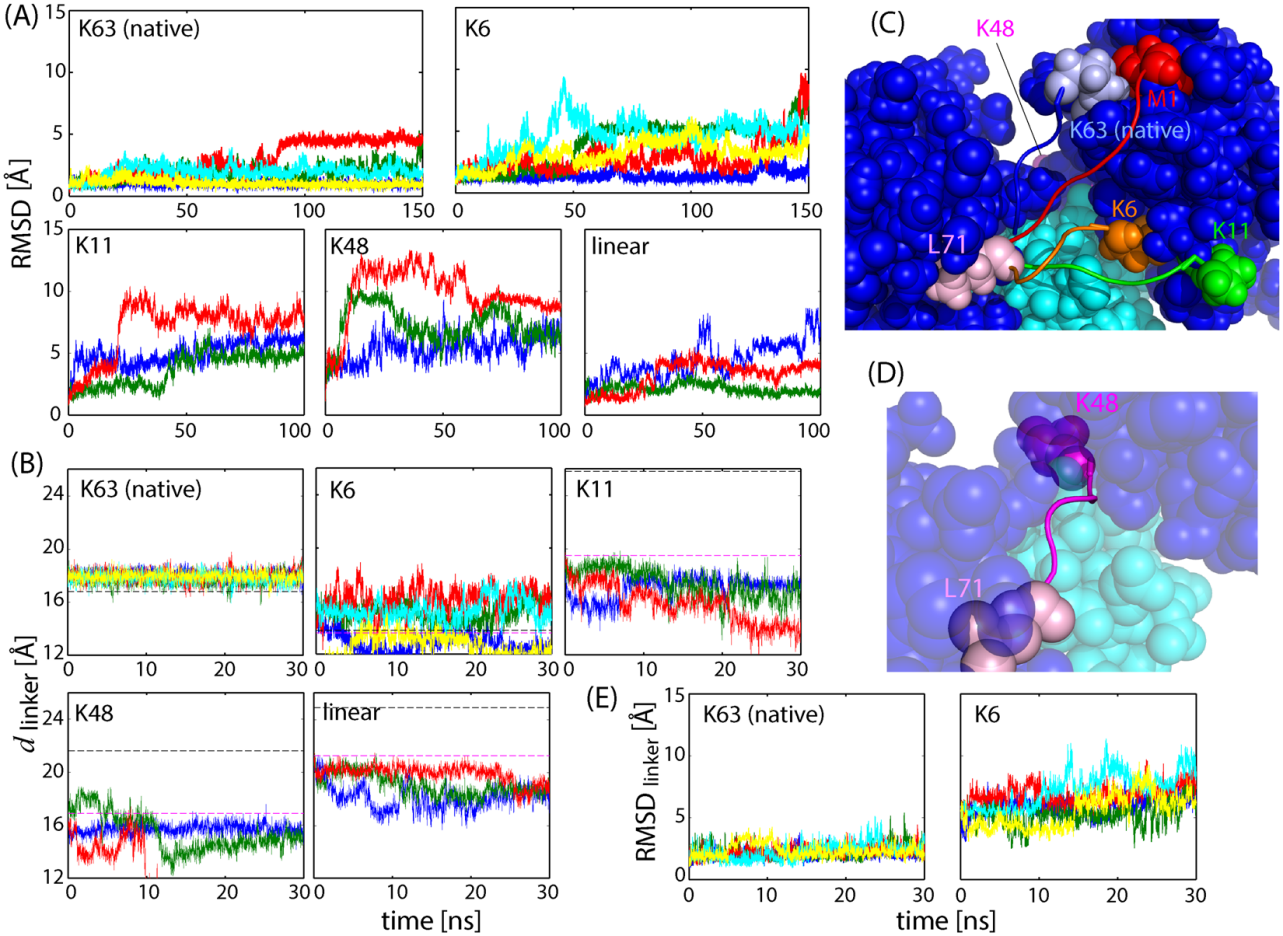
MD simulations of native K63-diUb/TAB2 NZF complex and artificially linked K6, K11, K48 di-UB/TAB2 NZF complexes. (**A**) Cα RMSD during the simulations of the five di-Ub/TAB2 NZF complexes (native K63: 5 × 150 ns; K6: 5 × 150 ns; K11, K48 and linear: 3 × 100 ns) from each initial model after superimposing Cα atoms of di-Ub (1–70). Note that the initial model for K63 is the crystal structure (PDB: 2wwz), but that the other models are different from each other depending on the modeling procedures (see Methods for details). (**B**) The linker length, *d*_linker_, or distance between Cα of L71 and N_ζ_ of each Lys residues (either K63, K6, K11, or K48 di-Ub) or N of M1 for linear di-Ub. The first 30 ns data are shown here. The horizontal black and pink lines are the *d*_linker_ values for the crystal structure and for the initial models, respectively; these values are different from each other depending on the linkage. (**C**) The initial models for MD simulations: di-Ub: blue; TAB2 NZF: cyan; linkers (71–76) and lysine residues (or M1): light blue (K63), red (linear), orange (K6), and green (K11). The linker of K48 (pink) is not drawn here because it is located on the other side of the proximal Ub. (**D**) The initial model for the K48 complex. (**E**) Cα RMSD of the Ub-Ub linker (71–76) for K63 and K6 di-Ub/TAB2 NZF.

As shown in Fig. 7A, any artificial linkage drastically reduces complex stability. The main cause of instability comes from the linker length, *d*_linker_ (Fig. 7B). Except for K6, the lengths of the artificial linkages estimated from the crystal structure were too large to connect the distal G76 to the proximal K11, K48, or M1 (black broken lines in Fig. 7B). Accordingly, the initial models for the simulations were built by constraining the two terminal atoms of the linkage, the carboxyl atom of distal G76 and N_ζ_ of the proximal Lys (or N of the proximal M1), to come close to each other by moving the linker and side-chain of the proximal Lys. The modeling results showed that although the linker lengths became much shorter than the values in the crystal structure (pink broken lines in Fig. 7B), the linkers were more extended, and the Lys side-chain conformations changed from those of the stable crystal structure (Fig. 7C). Moreover, the linker lengths of the initial models of linear and K11 were still larger than the length of the native K63 and followed by a further decrease during the simulation. These strains and changes in linker lengths may trigger deformation of the complex structures.

In the case of K48, although the linker length was shortened sufficiently by modeling, the complex was highly unstable. As shown in Fig. 7C,D, because the proximal K48 is located on the other side of di-Ub, the linker must pass through the narrow path encompassed by the two Ub moieties and NZF domain, and the K48 side-chain turns back to face the distal G76. Steric hindrance and strain of the K48 side-chain may cause instability. The experimental *K*_d_ value of K48 di-Ub/TAB2 NZF (663–693) is 29 μM^9^, suggesting that the binding mode of K48 uses an interacting surface other than that of K63.

In contrast, the linker length of K6 is much shorter than that of K63, but K6 is still unstable. To determine the cause of instability, we calculated the Cα RMSD of the linker, and found that the linker of K6 was much more flexible than that of K63 (Fig. 7E). This indicates that the linker of K63 has an optimal length to properly maintain the distance between the two Ubs. In contrast, the K6 linker has a much shorter length to yield large flexibility and thus instability.

In summary, the linker length of K63 is optimal for maintaining the interacting surface of K63 di-UB/TAB2 NZF.

## Concluding Remarks

Dynamic recognition in the complex, K63 di-Ub/TAB2 NZF, was illustrated by the molecular dynamics simulations together with enhanced sampling. The FES of the complex was spread over an extensive configurational space, demonstrating that the flexible Ub moieties loosely hold the NZF domain mostly in a bidentate manner. Nevertheless, the FES maintains a substantial population of the crystal structure of the complex as the minimum free-energy structure (ensemble close to the crystal structure occupies 14% of the whole ensemble; defined by RMSD TAB2 NZF < 4 Å and RMSD di-Ub < 2 Å; Fig. 2), which differs from the artificial complex, linear di-Ub/TAB2 NZF, whose FES exhibited an unstable monodentate binding mode with no stable structure as the funnel bottom. Despite the weak binding, the K63 linkage specificity to TAB2 NZF is achieved by the complemental interacting surfaces and proper length of the Ub-Ub linker.

Considering the correspondence between the FES (Fig. 2) and the experimental *K*_d_ value of a fluorescence anisotropy experiment, *K*_d_ = 8 μM for K63 di-Ub/TAB2 NZF^9^, it is noted that the *K*_d_ value reflects the contributions from numerous binding modes recognized as the bound state, not solely from the minimum energy structure corresponding to the crystal structure, because the fluorescence anisotropy detects the hydrodynamic property of the whole complex, not a specific structure. Suppose that only the structural ensemble close to the crystal structure (14% contribution to the whole ensemble) was regarded as the bound state, and if the other part of the ensemble was defined to be in the dissociated state, then *K*_d_ would increase by an order of magnitude. This explains how the dynamic recognition of the di-Ub complex with *K*_d_ = 8 μM differs from a protein complex with a similar *K*_d_ value characterized by a single binding mode. Although each binding mode is not sufficiently stable, an ensemble of the numerous binding modes allow the ligand molecule to stay around the receptor molecule with a sufficiently strong affinity.

The dynamic recognition does not yield a stable rigid complex structure like an enzyme-inhibitor complex^24^, but simply localizes a specific UBD around a poly-Ub chain according to the linkage specificity. As mentioned in the case of NF-κB, when the role of poly-Ub chains is to transiently colocalize several components of the signal transduction pathway at a specific cellular portion, the weak but specific binding of dynamic recognition is necessary and sufficient for the reaction in the cellular processes, which colocalizes the components but still allows for sufficient mobility required for the biochemical reactions. Recently, it was reported that poly-Ub (K63 or linear) induced liquid-liquid phase separation of the autophagy receptor p62^31^. The liquid-liquid phase separation, forming membrane-less liquid-like droplets in which various biochemical reactions occur, has been observed in a variety of cellular processes^32,33^. Considering that the liquid-like droplets are formed by flexible and multivalent protein-protein interactions, we predict that dynamic recognition of the di-Ub complex observed in the present study is an atomistic picture representing the poly-Ub-induced phase separation.

## Methods

### Simulation models

In this study, numerous MD simulations were performed for various di-UB/NZF complexes and mono-Ub/NZF complexes, as well as for free di-Ub, to evaluate linkage-specific recognition of the NZF domains. First, the simulation systems of the three native complexes, K63 di-Ub/TAB2 NZF, linear di-Ub/HOIL-1L NZF, and K33 di-Ub/TRABID NZF1, were constructed from the crystal structures of 2wwz (or 3a9j), 3b0a, and 5af6 in the protein data bank (PDB), respectively. The missing loops of the structures were modeled using MODELLER^34^. For K63 di-Ub/TAB2 NZF of 3a9j, the N-terminal tag of TAB2 NZF was removed to make the 665–693 construct. For linear di-Ub/HOIL-1L NZF, the HOIL-1L NZF Δtail (192–223) construct was mainly used, but HOIL-1L NZF containing the C-terminal helix (192–249) was also studied as a reference.

Second, the simulation systems of non-native complexes of linear di-Ub/TAB2 NZF and K33 di-Ub/TAB2 NZF were constructed by superimposing distal Ub of K63 di-Ub/TAB2 NZF (2wwz) to the distal Ub of each complex, and then replacing each native NZF domain with TAB2 NZF, respectively (Fig. 1E).

Third, the free di-ubiquitin systems, K63 di-Ub, linear di-Ub, and K33 di-Ub were constructed by removing the NZF domains from the complexes. The two mono-Ub/TAB2 NZF complexes, K63 distal-Ub/TAB2 NZF and K63 proximal-Ub/TAB2 NZF, as well as mono-Ub/NPL4 NZF, were studied. Their simulation systems were built by removing the proximal Ub and distal Ub from the complex, respectively, and mono-Ub/NPL4 NZF were based on the crystal structure, 1q5w.

Furthermore, simulation systems of artificial linkages of di-Ub/TAB2 NZF were constructed. The Ub-Ub linker of distal Ub (residue 71–76) was once removed from K63 di-Ub/TAB2 NZF and then MODELLER^34^ was used for remodeling so that the distal Ub bonded to one of the six Lys residues other than K63, or the N-terminal of M1, on the proximal Ub. The attempted model constructions resulted in successfully producing four different models with non-native linkages: K6, K11, K48, and linear (Fig. 7D,E).

For all simulation systems, rectangular simulation boxes were constructed with a margin of 10 Å to the boundary of the simulation box and filled by TIP3P water molecules^35^ and sodium/chloride ions so that the ion concentration reflected physiological conditions (200 mM) and to neutralize the simulation systems. The CHARMM 36 all-atom force field^36^ was used for the potential energy function. The isopeptide bonds between the N_ζ_ atoms of lysine (K63 and K33 on proximal Ub) and carboxyl atoms of glycine (G76 on distal Ub) were built and associated force field parameters were prepared by the molx module of the MD program MARBLE^37^.

### MD simulations

Simulations were performed with MARBLE^37^. Electrostatic interactions were calculated using the particle-mesh Ewald method^38^. The Lennard–Jones potential was smoothly switched to zero over the range of 8–10 Å. The symplectic integrator for rigid bodies was used to constrain the bond lengths and angles involving hydrogen atoms^37^, using a time step of up to 2.0 fs. During the equilibration phase, the systems were gradually heated to 300 K for 1 ns with gradually lower position-harmonic restraints for the proteins and then equilibrated at 300 K without restraints for 2 ns. The simulations were performed for the NPT ensemble at *T* = 300 K and *P* = 1 atm. Three to five production runs were carried out for each system described above (see Results in detail).

### MSES simulation

To evaluate the FES of the di-UB/NZF complexes in atomistic detail, the enhanced sampling simulations were performed using MSES. The calculation of the free energy landscape or the well-converged structural ensemble is essential for the comprehensive understanding of protein functional dynamics. However, it is difficult for a brute-force MD simulation to cover the whole configurational space related to the functional dynamics of large protein systems including explicit solvent. To overcome this problem, we have developed MSES, in which the sampling of the all-atom model is enhanced by coupling with accelerated dynamics of the associated coarse-grained (CG) model^20^. We have applied MSES to many protein systems of biological importance^23–26^ with methodological improvements^21,22^.

MSES adopts a multiscale simulation system comprising an all-atom model of proteins with surrounding solvents (**r**_MM_; *N* degrees of freedom) and the associated CG model (**r**_CG_; *M* degrees of freedom)^20–22^. The MSES potential energy is given by:

1 $$V={V}_{{\rm{MM}}}({{\bf{r}}}_{{\rm{MM}}})+{V}_{{\rm{CG}}}({{\bf{r}}}_{{\rm{CG}}})+{V}_{{\rm{MMCG}}}({{\bf{r}}}_{{\rm{MM}}},\,{{\bf{r}}}_{{\rm{CG}}},\,{k}_{{\rm{MMCG}}}),$$

with

2 $${V}_{{\rm{MMCG}}}={k}_{{\rm{MMCG}}}{[{{\boldsymbol{\chi }}}_{{\rm{MM}}}({{\bf{r}}}_{{\rm{MM}}})-{{\boldsymbol{\chi }}}_{{\rm{CG}}}({{\bf{r}}}_{{\rm{CG}}})]}^{2},$$

where *V*_MM_ and *V*_CG_ are the MM and CG potential energy functions, respectively. *V*_MMCG_ is the coupling term between MM and CG with a coupling constant, *k*_MMCG_. Herein, ${{\boldsymbol{\chi }}}_{{\rm{CG}}}({{\bf{r}}}_{{\rm{CG}}})$ is defined by *K* collective variables of CG coordinates, and ${{\boldsymbol{\chi }}}_{{\rm{MM}}}({{\bf{r}}}_{{\rm{MM}}})$ is a *K*-dimensional vector that is a projection of **r**_MM_ onto the associated *K*-dimensional space. Thus, *V*_MMCG_ is used to efficiently accelerate the MM dynamics via coupling with CG.

The intrinsic free energy surface solely from *V*_MM_ can be obtained by eliminating bias from the coupling *V*_MMCG_. To do this, Hamiltonian replica exchange^39,40^ was adopted, in which many replicated systems were assigned with various values of *k*_MMCG_ from a large value to zero; then, the structures with *k*_MMCG_ = 0 is the target ensemble. The exchange probability between replicas *m* and *n*, satisfying the detailed balance condition, is given by:

3 $${p}_{mn}=\,{\rm{\min }}(1,\,\exp ({{\rm{\Delta }}}_{mn})),$$

with

4 $${{\rm{\Delta }}}_{mn}=\beta ({k}_{{\rm{MMCG}}}^{m}-{k}_{{\rm{MMCG}}}^{n})({V}_{{\rm{MMCG}}}^{m}-{V}_{{\rm{MMCG}}}^{n}),$$

where *β* is the inverse temperature of the system. The condition *K* < *M* ≪ *N* ensures a low value of Δ_*mn*_, and high exchange probability *p*_*mn*_ irrespective of the size of MM, *N*. This guarantees excellent scalability that is applicable to large protein systems in solution. Furthermore, the approximation of adiabatic separation was used for efficient sampling by making CG much heavier than MM to slow the CG motion, while making the CG temperature, 1/*β*′, much higher than 1/*β* of MM; i.e., *β*′ ≪ *β*^41–45^. Under adiabatic separation, MSES consists of replicated MM systems with various *k*_MMCG_ values (Eq. 2) and a single copy of CG that does not feel counterforce from MM, subjected to Markov chain Monte Carlo with the exchange probability, *p*_*mn*_ (Eq. 3).

MSES simulations were successfully performed for the native K63 di-Ub/TAB2 NZF and non-native linear di-Ub/TAB2 NZF complexes. The all-atom potential energy *V*_MM_ was the same as that described previously. For the CG models, the Cα atoms of K63 di-Ub and TAB2 NZF were chosen as coordinates, i.e., *M* = (152 + 32) atoms × 3. The CG potential energy *V*_CG_ was set as the sum of two terms to represent the intra-protein interactions and protein-protein interactions. For intra-protein interactions, the elastic network model^46^ was adopted together with a force constant of 0.75 kcal/mol/Å^2^ and cut-off length of 12 Å. For protein-protein interactions, the 6–12 Lennard-Jones potential was employed for 123/108 pairs (K63/M1) of the Cα atoms between the di-ubiquitin and TAB2 NZF together with a soft harmonic boundary to avoid overly large separation between the two proteins, as in our previous studies^24,25^. These Cα atom pairs were selected as they were located within 15 Å in the starting structures for the simulations. The minimum depth and distance for the LJ potential were set respectively as 0.03 kcal/mol and the distances in the starting structures. The soft boundary potential started at 12 Å apart from the minimum of the LJ potential, with a harmonic force constant of 5 kcal/mol/Å^2^. Under conditions of adiabatic separation, the mass of the CG atom was set to 1 × 10^4^ amu (atom mass unit). CG simulation was performed by using Berendsen’s thermostat under the constant temperature (NVT) condition at *T* = 1 × 10^3^ K using μ^2^lib (http://www.mu2lib.org/).

For the coupling potential *V*_MMCG_, *K* = 123/108 pairs of Cα atoms between di-ubiquitin and TAB2 NZF were used as variables of χ_MM_ and χ_CG_, which were chosen according to the condition that their distances were less than 12 Å in the starting structures. The Markov chain Monte Carlo calculations were carried out every 20 ps using 16 replicas with *k*_MMCG_ = 0, 0.0002, 0.0005, 0.0011, 0.0021, 0.0037, 0.006, 0.0092, 0.0135, 0.0192, 0.0265, 0.0357, 0.0471, 0.0611, 0.078, and 0.098 kcal/mol/Å^2^. The number of iterations for each MSES simulation was 10,000, corresponding to 200 ns simulation and 16 replicas × 200 ns × 2 simulations = 6.4 μs in total. MSES simulations were performed using our script file, which is available from the authors upon request.

## Electronic supplementary material

Supplementary Material

## Data Availability

The datasets generated during and/or analysed during the current study are available from the corresponding author on reasonable request.
