# Supplementary material for: Dynamic recognition and linkage specificity in K63 di-ubiquitin and TAB2 NZF domain complex
Source: Sci Rep. 2018 Nov 7;8:16478. doi: 10.1038/s41598-018-34605-2 (PMC6220233; doi:10.1038/s41598-018-34605-2)
Supplement: Supplementary file 1 — Supplementary Material [file 41598_2018_34605_MOESM1_ESM.pdf]

Supplementary Material

Dynamic recognition and linkage specificity in K63 di-ubiquitin and TAB2 NZF domain complex

Kei Moritsugu, Hafumi Nishi, Keiichi Inariyama,
Masanori Kobayashi & Akinori Kidera

Graduate School of Medical Life Science, Yokohama City University,
1-7-29 Suehiro-cho, Tsurumi-ku, Yokohama 230-0045, Japan

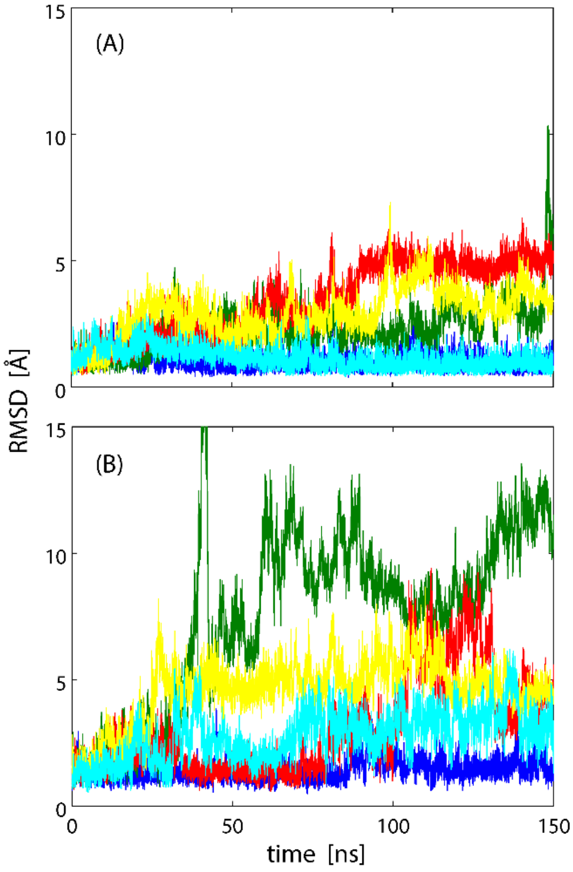

Figure S1. MD simulations of K63 di-Ubs/TAB2 NZF complex.
(A) Time courses of Cα RMSD values for five 150-ns MD simulations (colored in red, blue, green, cyan, and yellow) of K63 di-Ubs/TAB2 NZF complex (PDB: 2wwz; TAB2 NZF (662–693)). (B) Those of K63 di-Ubs/TAB2 NZF complex (PDB: 3a9j; TAB2 NZF (665–693 lacking the N-terminal acidic residues).

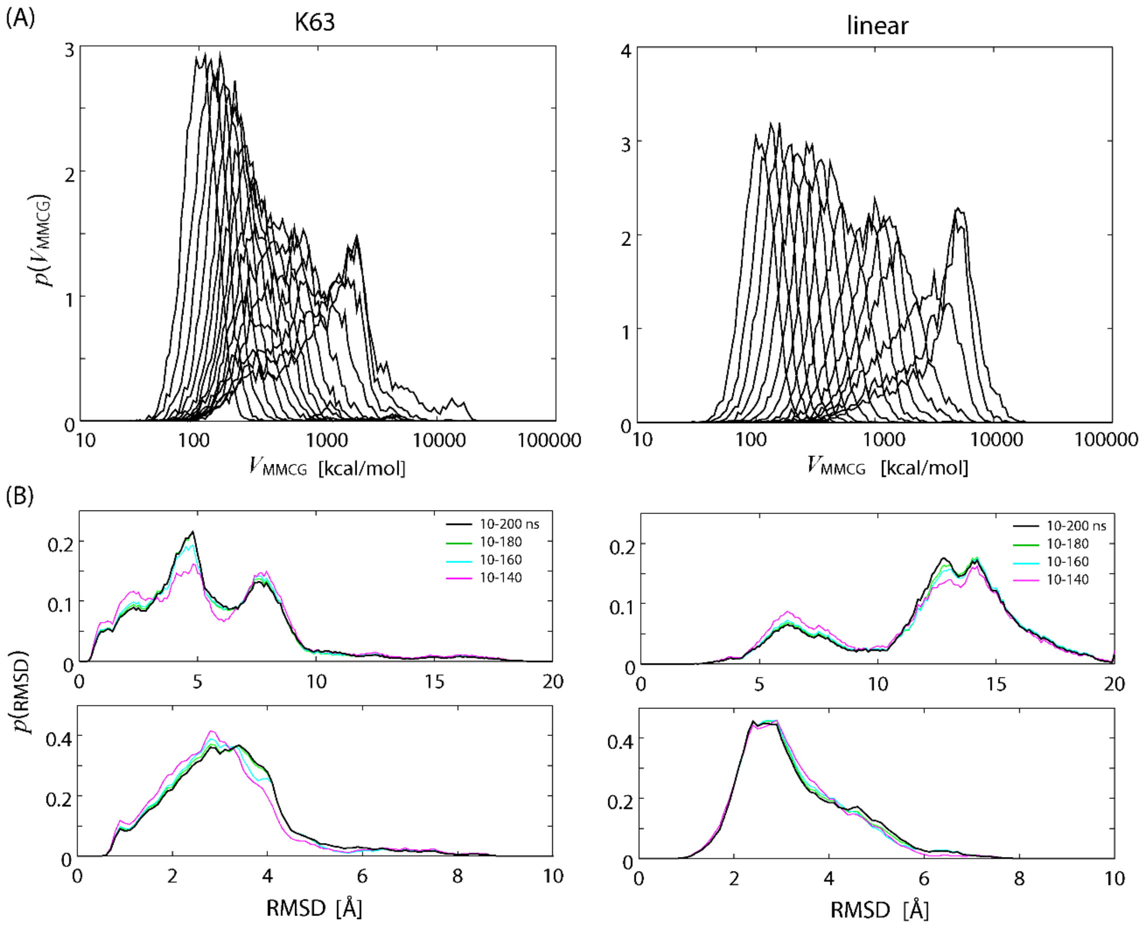

Figure S2. MSES simulations.
(A) Energy overlap of V_{MMCG} for 16 replicas in the Hamiltonian exchanges. (B) The convergence of MSES simulations. The upper and lower distributions correspond to the values along the x-axis and y-axis of Fig. 2A, respectively. MSES simulations of K63 di-Ub/TAB2 NZF (left) and linear di-Ub/TAB2 NZF (right).

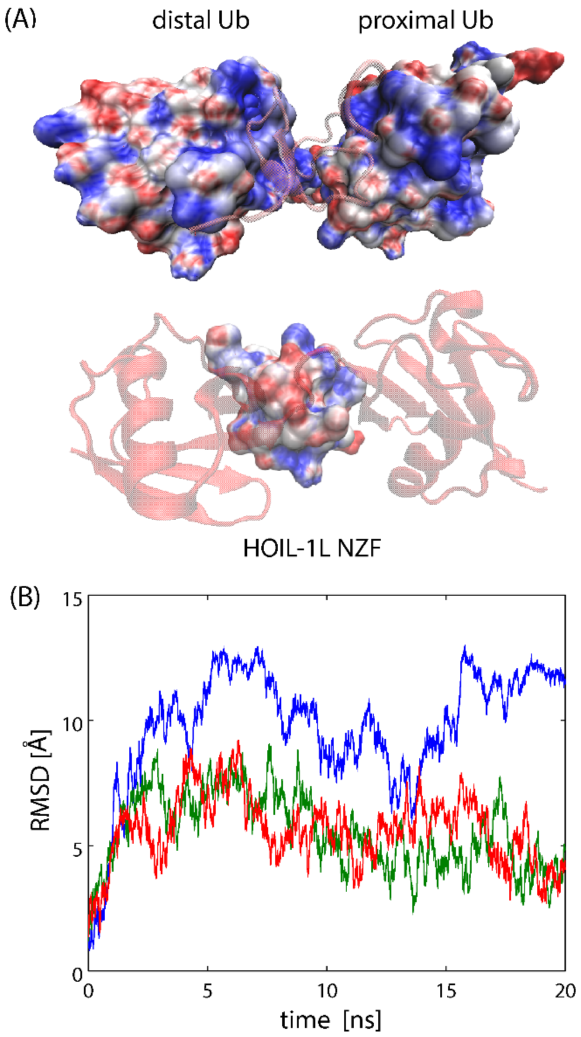

Figure S3. Electrostatic interactions between linear di-UB and HOIL-1L NZF. (A) Surfaces of the electrostatic potential of linear di-Ub without HOIL-1L NZF and isolated HOIL-1L NZF, drawn by VMD[45]. (B) Time courses of Cα RMSD values for three MD simulations (colored in red, blue, and green) of free linear di-Ub starting from the crystal structure of the complex after removing HOIL-1L NZF.

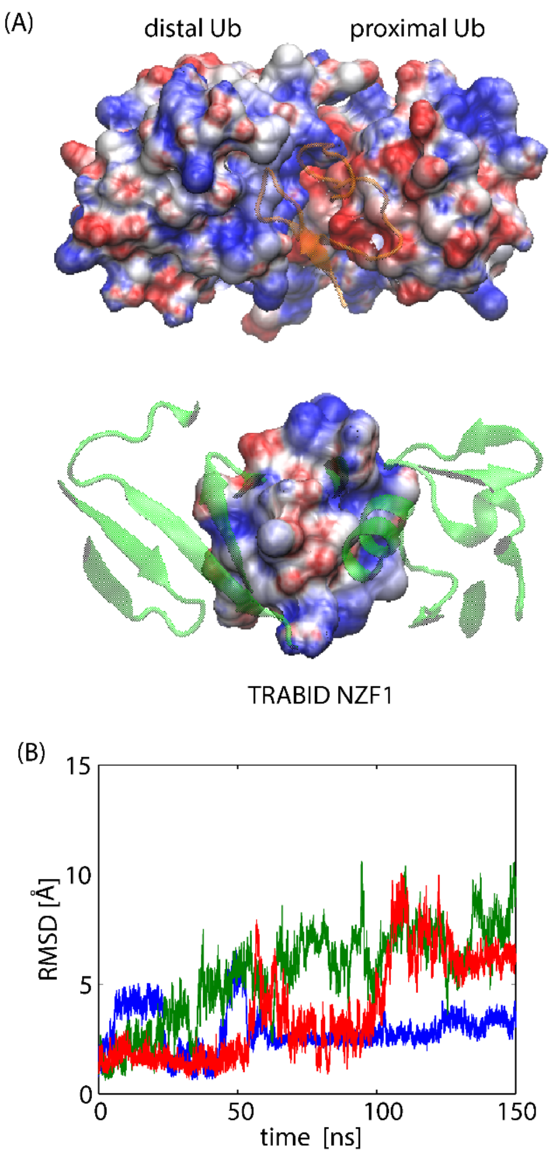

Figure S4. Electrostatic interactions between K33 di-UB and TRABID NZF1. (A) Surfaces of the electrostatic potential of K33 di-Ub without TRABID NZF1 and isolated TRABID NZF1, drawn by VMD[45]. (B) Time courses of Cα RMSD values for three MD simulations of K33 di-Ub (colored in red, blue, and green) starting from the crystal structure of the complex after removing TRABID NZF1.

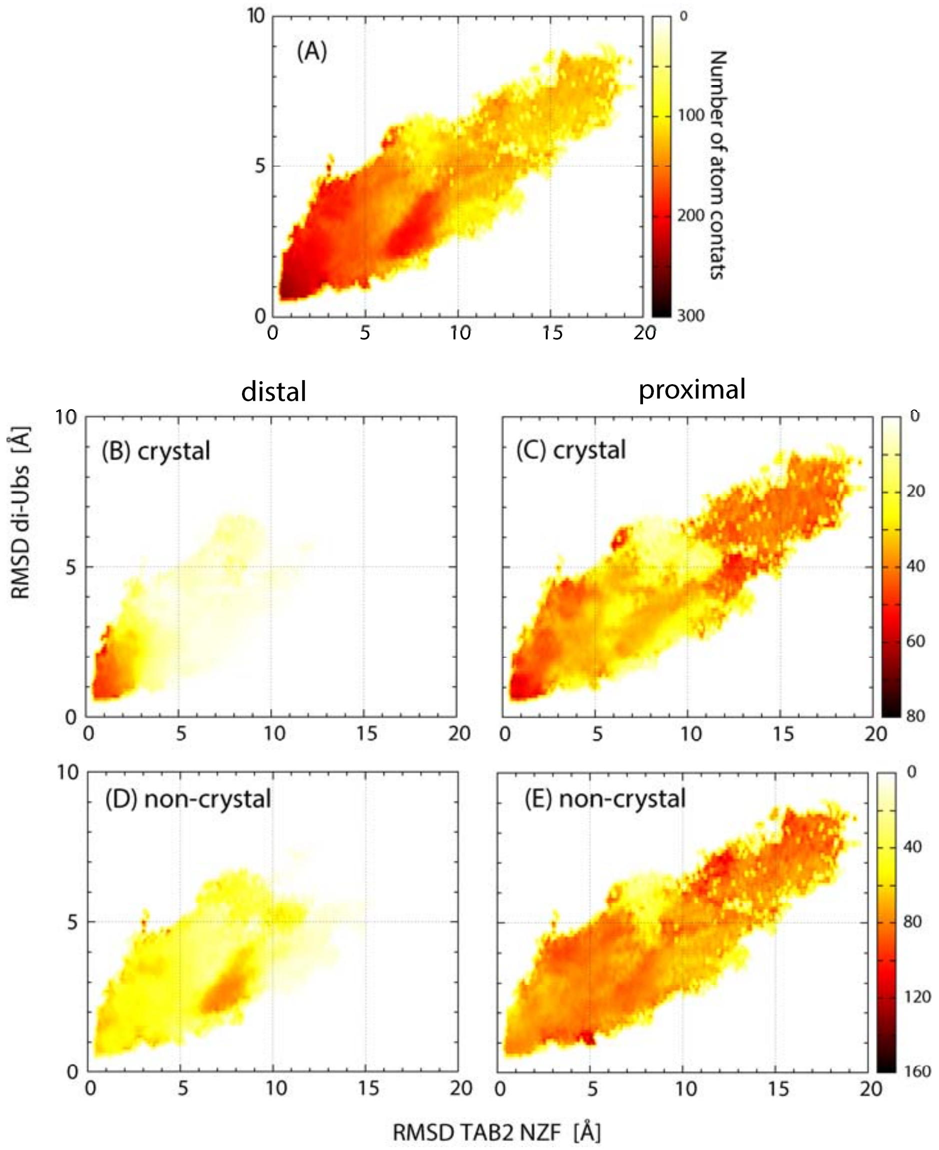

Figure S5. FES viewed from the contacts in K63 di-Ubs/TAB2 NZF.
Same as Fig. 2A, but the number of inter-molecular contacts between Ubs and TAB2 NZF is plotted rather than free energy. Contacts (A) for the overall, (B) found in the crystal structure on the distal Ub, (C) found in the crystal structure on the proximal Ub, (D) found only in the simulation on the distal Ub, and (E) found only in the simulation on the proximal Ub. The distal Ub has contacts only in the near-native ensemble, while the proximal Ub has contacts both in the near-native and non-native ensembles.

```
TAB2_HUMAN 661 EDDEGAQWNCTACTFLNHPALIRCEQCEMPRHF  693
TAB2_MOUSE 661 EDEEGAQWNCTACTFLNHPALIRCEQCEMPRHF  693
TAB2_DANRE 679 EEDDGVQWSCTACTFLNHPALNRCEECEFPRNF  711
TAB3_HUMAN 680 EDYEGAPWNCDSCTFLNHPALNRCEQCEMPRYT  712
TAB3_XENLA 660 EDFEGSPWNCNSCTFLNHPALNRCEQCEMPRFT  692
```

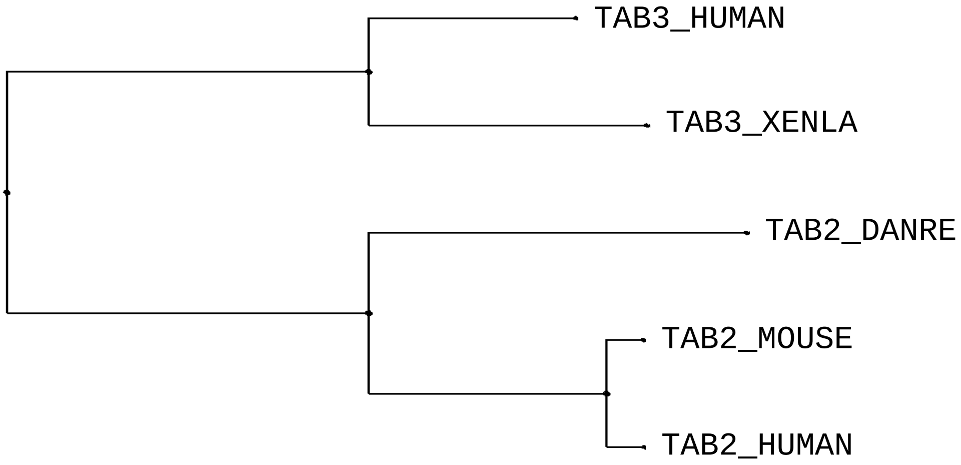

Figure S6. Sequence alignment and phylogenetic tree of the TAB2 and TAB3 NZF domains.
The common amino acids are colored in red. The N-terminal acidic residues are indicated by a red box.

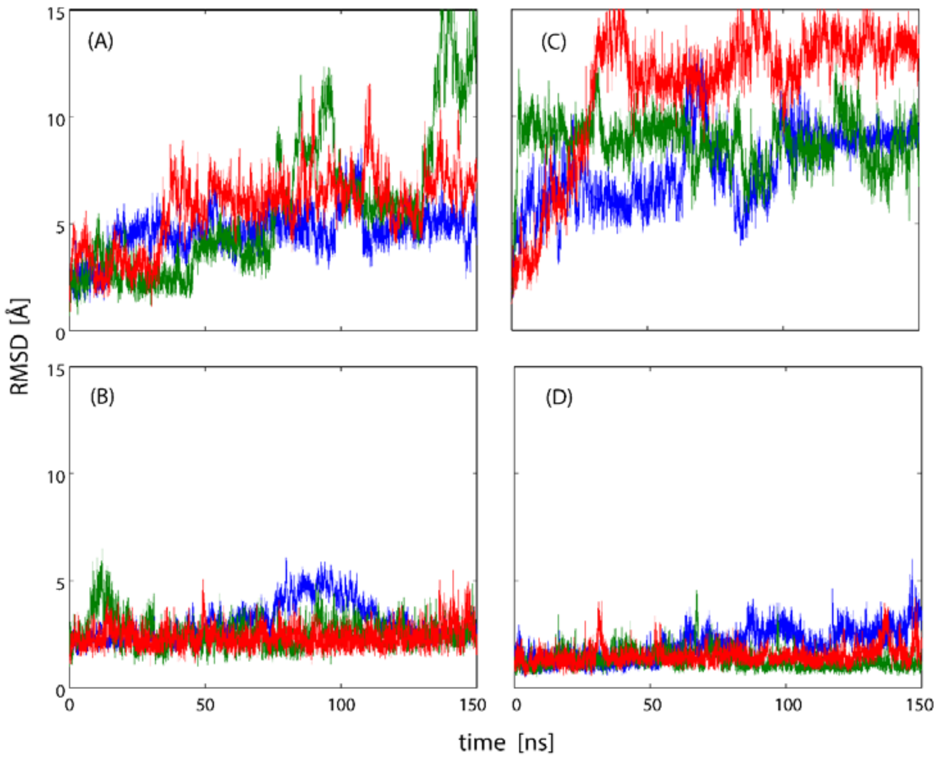

Figure S7. MD simulations of linear di-Ub/HOIL-1L NZF, linear di-Ub/HOIL-1L, and K33 di-Ub/TRABID NZF1.

Time courses of Cα RMSD values for the three MD simulations (colored in red, blue, and green). (A) Linear di-Ub/HOIL-1L NZF (192–223) from the crystal structure (PDB: 3b0a). (B) Linear di-Ub/HOIL-1L NZF whose NZF domain contains the C-terminal helix (NZF (192–249)). (C) Linear di-Ub/TAB2 NZF from the initial homology model. Same as that of Fig. 6A–C, but obtained from MD simulations rather than MSES simulation. (D) K33 di-Ub/TRABID NZF1 from the crystal structure (PDB: 5af6).

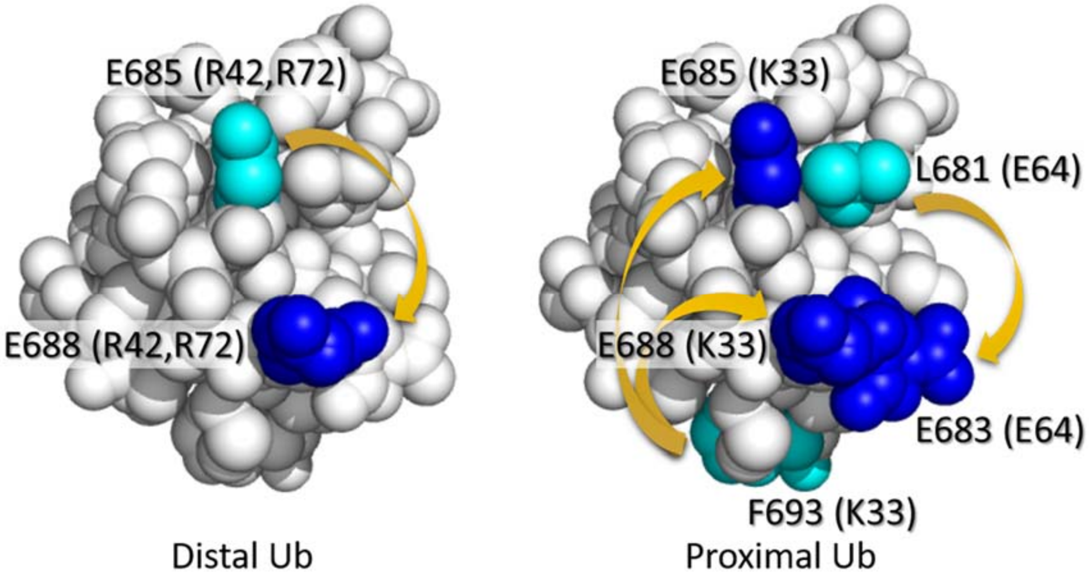

Figure S8. Salt bridges of TAB2 NZF in complex with linear di-Ub.
The salt bridges in the native linear di-Ub/HOIL-1L NZF are colored in cyan, while those in linear di-Ub/TAB2 NZF are in blue. The residue numbers are those of TAB2 NZF and those in parentheses are the binding partners of Ubs.

Table S1. Probability of inter-molecular residue contacts for K63 di-Ub/TAB2 NZF.

polar contacts

Ub	TAB2 NZF	$P_{contact}$[a,b]
distal		
R42	Q686	0.28
G47[c]	L676	0.29
Q49	Q686	0.27
R72	E685	0.42
R72	Q686	0.23
R72	E688	0.41
proximal		
K6	E688	0.46
R42	D662	0.21
R42	E664	0.73
A46	E685	0.20
G47	Q667	0.40
R72	D662	0.44

non-polar contacts

Ub	TAB2 NZF	$P_{contact}$
distal		
I44	F675	0.43
I44	Q686	0.44
G47	F675	0.42
V70	Q686	0.53
proximal		
L8	A680	0.64
L8	L681	0.76
L8	I682	0.69
R42	H678	0.54
R42	L681	0.41
I44	H678	0.93
I44	L681	0.96
G47	L676	0.53
G47	H678	0.69
H68	L681	0.94
H68	E685	0.42
V70	A680	0.95
V70	L681	0.92

[a] Probability of occurrence of inter-molecular residue contacts during MSES simulation was calculated for residue pairs, when any atom pair of a residue in Ub and the other residue in NZF formed a contact. A polar contact was defined as a hydrogen bond if the nitrogen-carbon distance was less than 3.5 Å. A non-polar contact was defined by the distance of the non-hydrogen atoms <4.0 Å.
[b] $P_{contact}$ >0.2 for polar contacts and >0.4 for non-polar contacts are listed.
[c] Residue pairs colored in red are those found in the crystal structure. Classification was conducted on the residue-base.

Table S2. Probability of inter-molecular residue contacts for linear di-Ub/HOIL1-L NZF and linear di-Ub/TAB2 NZF.

polar contacts

Ub	HOIL-1L NZF	$P_{contact}$[a,b]	TAB2 NZF	$P_{contact}$
distal				
R42	E212	0.21	E685	
R42	C215		E688	0.47
G47[c]	I203	0.21	L676	
R72	E212	0.61	E685	
R72	M213	0.62	Q686	0.22
R72	C215		E688	0.65
proximal				
Q2	R208	0.43	L681	
Q2	C211	0.27	C684	
K11	E220	0.35	F693	
K33	E212		E685	0.26
K33	C215		E688	0.22
K33	E220	0.26	F693	
K63	C215		E688	0.23
E64	R208	0.79	L681	
E64	G210		R683	0.42

nonpolar contacts

Ub	HOIL-1L NZF	$P_{contact}$	TAB2 NZF	$P_{contact}$
distal				
L8	C214	0.42	C687	
L8	R216	0.46	M689	
I44	C200	0.45	C673	
I44	F202	0.56	F675	
I44	M213	0.82	Q686	
G47	T201	0.74	T674	
G47	F202	0.73	F675	
Q49	M213	0.51	Q686	
V70	M213	0.88	Q686	
V70	C214	0.67	C687	
R72	C215	0.51	E688	
proximal				
Q2	C215	0.76	E688	
F4	R208	0.50	L681	
F4	P209	0.57	I682	
T12	P209	0.76	I682	
T14	P209	0.42	I682	
T14	G210	0.56	R683	
T14	C215	0.82	E688	

[a] Same as Table S1. The data of linear di-Ubs/HOIL1-L NZF were obtained from three 150-ns MD simulations, and those of linear di-Ubs/TAB2 NZF from MSES simulation. Therefore, the comparison of the absolute values is not significant. The residues on the same row are corresponding amino acids in the alignment of Fig. 1E.
[b] Same as Table S1.
[c] Same as Table S1.

Table S3. Probability of inter-molecular residue contacts for K33 di-Ub/TRABID NZF1 and K33 di-Ub/TAB2 NZF

polar contacts

Ub	TRABID NZF	$P_{contact}$[a,b]	TAB2 NZF	$P_{contact}$
distal				
R42	M26		Q686	0.22
G47[c]	E16	0.72	L676	
R72	T25	0.76	E685	
R72	M26	0.88	Q686	0.25
R72	R28		E688	0.23
proximal				
E18	K23	0.75	R683	0.38
E18	R28	0.41	E688	
D21	R28	0.76	E688	
D21	K23		R683	0.56
K29	R28		E688	0.25

nonpolar contacts

Ub	TRABID NZF	$P_{contact}$	TAB2 NZF	$P_{contact}$
distal				
L8	Y12	0.94	A672	
L8	C13	0.79	C673	
L8	C27	0.72	C687	
R42	M26	0.62	Q686	
I44	C13	0.50	C673	
I44	T14	0.98	T674	
I44	Y15	0.97	F675	
I44	M26	0.85	Q686	
G47	T14	0.77	T674	
G47	Y15	0.90	F675	
Q49	Y15	0.50	F675	
Q49	M26	0.66	Q686	
H68	C13	0.71	C673	
H68	T14	0.98	T674	
V70	C13	0.85	C673	
V70	M26	0.99	Q686	
V70	C27	0.63	C687	
V70	R31		R691	0.46
proximal				
S20	I22	0.74	A672	
T22	W18	0.71	H678	
T22	S20	0.68	A680	
T22	A21	0.97	L681	
T22	T25	0.43	E685	
E24	Y15	0.61	F675	
E24	W18	0.78	H678	
E24	T25	0.89	E685	
N25	C24	0.47	C684	
N25	T25	1.00	E685	
N25	R28	0.78	E688	
A28	T25	0.79	E685	
D52	W18	0.43	H678	
G53	W18	0.96	H678	
G53	S20	0.80	A680	
T55	S20	0.92	A680	

[a] Same as Table S1. The data of K33 di-Ubs/ TRABID NZF1 and K33 di-Ubs/ TAB2 NZF were obtained from three 150-ns MD simulations. The residues in the same row are corresponding amino acids in the alignment of Fig. 1E.
[b] Same as Table S1.
[c] Same as Table S1.

Table S4. Probability of inter-molecular polar contacts for the proximal Ub type complex of mono-Ub/TAB2 NZF.

polar contacts

mono-Ub	TAB2 NZF	$P_{contact}^{a,b}$
K6	E688	0.48
R42[c]	D662	0.21
R42	E664	0.49
A46	E685	0.35
G47	Q667	0.29
G47	E685	0.36
R72	D663	0.21
R74	D662	0.21
R74	D663	0.23

[a] Same as Table S1. The data were obtained from three 150-ns MD simulations.
[b] Same as Table S1.
[c] Same as Table S1.
